# Transplantation of fetal liver tissue coated by ultra-purified alginate gel over liver improves hepatic function in the cirrhosis rat model

**DOI:** 10.1038/s41598-020-65069-y

**Published:** 2020-05-19

**Authors:** Rong Qiu, Soichiro Murata, Katsutomo Oshiro, Yumi Hatada, Hideki Taniguchi

**Affiliations:** 1 Department of Regenerative Medicine, Yokohama City University Graduate School of Medicine, 3-9, Fuku-ura, Kanazawa-ku, Yokohama, Kanagawa 236-0004 Japan; 2 Division of Regenerative Medicine, Center for Stem Cell Biology and Regenerative Medicine, the Institute of Medical Science, the University of Tokyo, 4-6-1, Shirokanedai, Minato-ku, Tokyo 108-8639 Japan

**Keywords:** Hepatocytes, Regenerative medicine

## Abstract

In this study, we used a new coating agent, that is, ultra-purified alginate gel (UPAL), for fetal liver tissue transplantation. This study aims to compare the effect of UPAL with the effect of other coating agents on improving the effect of fetal liver tissue transplantation in a liver cirrhosis rat model. Prior to the transplantation of wild-type ED14 fetal liver tissues, various coating agents were separately applied on the liver surface of rats with cirrhosis. Then, we compared the engraftment area, engraftment rate and liver function level of these rats. As a result, coating the liver surface of a cirrhosis rat with UPAL obtained the best effect in terms of engraftment area and engraftment rate of the transplanted liver tissue and in the recovery of liver function compared with control group. Therefore, UPAL coating may serve as a novel strategy for liver organoid transplantation.

## Introduction

Liver cirrhosis is a condition in which the liver undergoes a slow scarring process due to long-term, repeated injury caused by liver diseases and/or particular conditions. Its major causes are chronic hepatitis B and C virus infection, alcoholic liver disease and nonalcoholic fatty liver disease^1,2^. Some pathological features of liver cirrhosis include liver tissue inflammation and necrosis, ascites and fibrosis at the anterior part of the liver^3,4^. Annually, one million people die due to liver cirrhosis complications worldwide^5^. Furthermore, patients with liver cirrhosis have a higher risk of liver cancer^1,6,7^. To date, liver transplantation has remained the only common treatment for end-stage liver cirrhosis. Additionally, shortage of donor liver limits the end-stage cirrhosis treatment^8^.

Several alternative treatments, such as medication, bone marrow cell^9^ transplantation and adipose tissue-derived stromal cell^10^ (collected from the patient) transplantation, have been proposed to treat end-stage liver cirrhosis. However, none of these treatments shows similar efficacy to the traditional liver transplantation method. Bone marrow cell transplantation somehow manages to improve liver function, but the transplanted cells failed to differentiate into fully functioned liver tissues^9^. Meanwhile, some medicines, such as PRI-724 (a CBP/β-Catenin inhibitor), are proven effective in improving liver histology and Child-Pugh scores in several patients with hepatitis C virus-related cirrhosis, but they still cannot cure the disease^11^.

Recovery of liver function in the living body by cell transplantation is possible. Cell and organ transplants can be applied into the cirrhotic liver by the portal vein, which is the mainstream of the liver. However, the portal vein injection of excessive number of cells might cause lethal portal embolism and liver necrosis^12^. In patients with cirrhosis, haemorrhage is caused by the reduction of blood coagulation factors and embolism of transplanted cells by the transfer of transplanted cells to other organs through the intrahepatic portal vein shunt. Considering the high risk of cell transplantation complications, such as portal vein embolism, applying portal vein grafting in the liver cirrhosis is difficult. Therefore, we have been attempting to discover a new transplantation method for liver cirrhosis. We established a method of implanting a fetal liver tissue^13^ between the middle lobe and the left lobe of liver fibrosis model for transplantation. However, using the same method is difficult because rodents and humans have some different anatomical structures of the liver. Therefore, we need to establish a transplant site and a method that can be applied clinically. Rat models of this disease are valuable for increasing our understanding of liver fibrosis.

Dipeptidyl peptidase-IV (DPPIV) is a membrane-associated peptidase, also known as CD26, that is widely used as marker for hepatocytes in several liver diseases, such as primary biliary cirrhosis and hepatocellular carcinoma (HCC)^14,15^. First, we established a cirrhosis rat model by administering dimethylnitrosamine (DMN) to dipeptidyl peptidase-IV (DPPIV)(−) F344 rats. DMN may cause liver parenchyma destruction, eventually leading to collagen deposition by activated stellate cells in the liver that resembles fibrosis as in cirrhosis. Next, we used a 14-day fetal (ED14) DPPIV(+) F334 rat liver tissue, which is similar to human iPSC liver buds, as a transplant tissue. Finally, different coating materials were used to cover the transplanted fetal rat liver tissue.

To assess the effect of coating materials, we measured engraftment rate, engraftment area and the expression of liver function marker. Then, we measured the liver function and survival rate with or without fetal liver tissue transplantation which coated by UPAL. In conclusion, the use of UPAL has the best effect in all of the coating materials and increase the recipient rat survival rate and liver function level compared with control group. Therefore, UPAL coating may serve as a novel strategy for liver organoid transplantation.

## Results

### Histological sections stained with Sirius red and liver function parameters of the DMN-induced liver cirrhosis in rats

**Figure 1:**
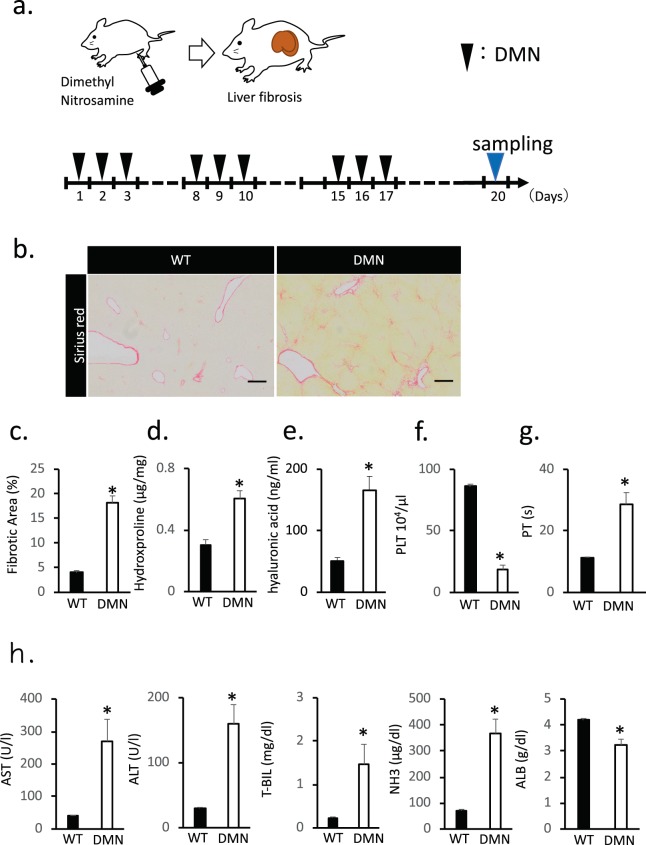
Histological sections stained with Sirius red and liver function parameters during the DMN-induced hepatic fibrosis in rats. (**a**) Schematic representation of the protocol for generating liver cirrhosis rats. After 20 days of injection, the livers were collected and analyzed. (**b**) Histological analysis of liver tissue from DMN-injected rats. Collagen stained with Sirius red is shown in red. Scale bar: 500 µm. (**c**) Fibrotic area quantified with an image analysis system and shown as percentage of the total area. (**d,e**) Hydroxyproline and hyaluronic acid levels in the liver fibrosis rats measured by ELISA at 20 days after DMN injection. (**f,g**) Concentrations of the blood PLT and PT level of rats assessed after DMN administration. (**h**) Measurement of the liver function indices (AST, ALT, T-BIL, NH_3_ and ALB) of rats after DMN injection. Data are mean ± SE. *P < 0.05, vs. wild-type Mann-Whitney *U* test, n = 6.

Liver cirrhosis was induced on the DPPIV(−) F344 rats by intraperitoneal administration of DMN for 3 weeks (Fig. 1a). DMN-induced liver fibrosis in rats was assessed histologically by Sirius red staining, which marked the fibrosis region by labelling the collagen fibrils. The fibrosis region marked by Sirius red staining was 5% of the total area in control rats’ liver and 20% in the DMN-treated rats’ liver. Sirius red staining values were consistently high for the DMN-treated group, which showed high fibrosis in the liver lobules and cellular structures, compared with that for the control group (Fig. 1b,c). We also assessed the collagen in the liver tissue by measuring the hydroxyproline level. Serum hyaluronic acid correlated with the degree of liver fibrosis. The liver hydroxyproline and serum hyaluronic acid levels increased corresponding to the liver fibrosis rate (*P* < 0.05) (Fig. 1d,e). The platelet (PLT) and prothrombin time (PT) levels were significantly different between the control and coated groups. In rats treated with DMN, the PLT levels decreased, whereas the PT levels increased (Fig. 1f,g). Figure 1h shows that a difference was observed in the major liver function indices, namely, serum aspartate transaminase (AST), alanine transaminase (ALT), total bilirubin (T-BIL) and NH_3_, between the control and DMN-treated rats. These indices in serum significantly increased after DMN administration. Conversely, the serum albumin (ALB) expression decreased in the DMN-treated rats (Fig. 1h). Therefore, DMN treatment caused liver cirrhosis in rats, thereby successfully establishing a cirrhosis rat model.

### Transplantation methods of fetal liver tissue onto the liver surface

**Figure 2:**
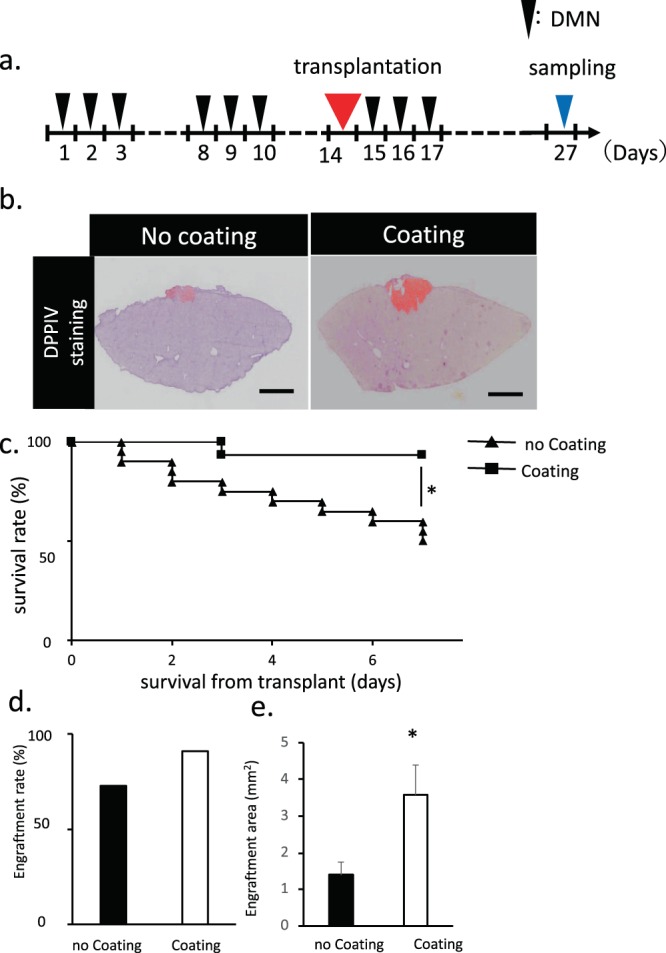
Transplantation methods of fetal liver tissue onto the liver surface. (**a**) Schematic of the transplantation of a fetal liver tissue coated with a coating agent. After 27 days of first DMN injection, the samples were collected and analyzed. (**b**) Histological analysis of liver tissue with a coating agent covering the liver surface before transplantation. DPPIV-stained living tissue is colored in red. Scale bar: 2 mm. (**c**) Measurement of the survival rate of fetal liver tissue transplantation after coating agent utilization. The survival rate of the transplanted group without coating agent was also monitored as a control. Data are mean ± SE. *P < 0.01, log rank test; no coating: n = 20, coating: n = 15. (**d,e**) Measurement of the engraftment rate and area of fetal liver tissue transplantation after coating agent utilization. The engraftment rate and area in the transplanted group without coating agent was also monitored as a control. Data are mean ± SE. *P < 0.05, vs. wild-type Mann-Whitney *U* test, n = 11.

After establishing a decompensated liver cirrhosis rat model, we attempted to establish a method for transplanting the ED14 rat fetal liver tissue onto the liver surface with fibrosis (Fig. 2a and Supplementary Video 1). The serous membrane on the middle lobe surface of the fibrotic liver was exfoliated, and the ED14 rat fetal liver tissue was transplanted on the serosal exfoliation surface. Then, we compared the changes observed between the Matrigel-coated group and the noncoated group.

To compare the transplantation efficiencies and the engraftment rate of the fetal liver tissue for the liver surface, we transplanted the same amount of fetal liver tissues. Engraftment rate was confirmed by DPPIV staining with or without coating. The engraftment rate of the transplanted fetal liver tissue significantly increased when the coating agent was used prior to transplantation (Fig. 2b). After 1 week of monitoring following transplantation, the survival rates of liver-transplanted rats were significantly higher in the coated group than in the noncoated group (Fig. 2c). Likewise, the engraftment rate in the coated group was higher than in the noncoated group. The engraftment rate was identical to the graft survival of the transplanted fetal liver tissues. Furthermore, the engraftment area was increased using the coating agent during transplantation (Fig. 2d,e). The living tissue amount, survival rate, engraftment rate and engraftment area gradually increased in the coated group, indicating that coating and fixing the transplanted tissue are necessary.

### Histological assessment of the transplanted fetal liver tissue in various coating agent groups

**Figure 3:**
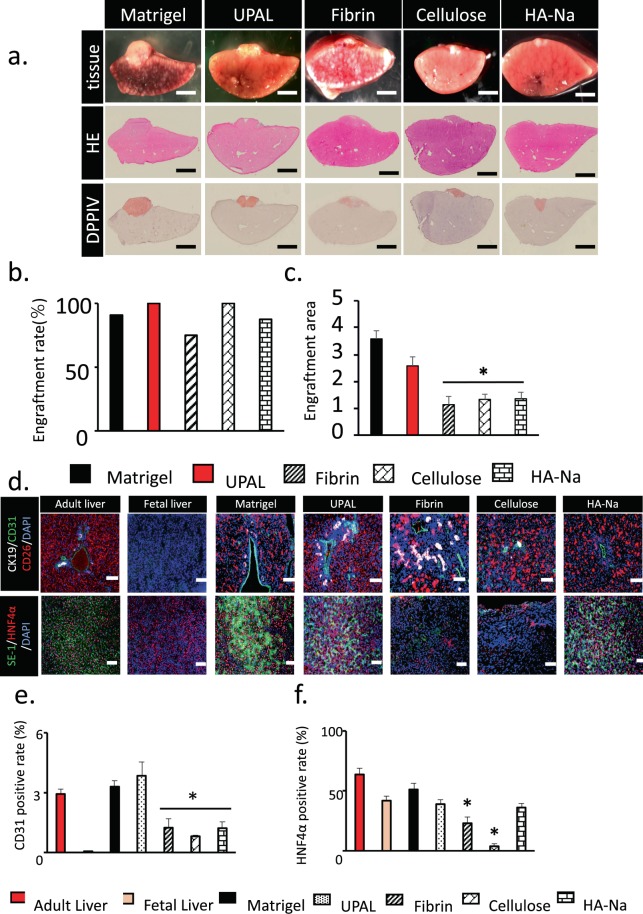
Histological assessment of the transplanted fetal liver tissue in five coating agent groups. (**a**) Histological analysis of the fetal liver tissue that was engrafted on the surface of the middle lobe of the liver and covered by a coating agent. HE staining of liver tissues onto the liver surface. DPPIV-stained living tissue is shown in red. Scale bar: 2 mm. (**b,c**) Measurement of the engraftment rate and area of transplantation of fetal liver tissue after coating agent utilization. Many kinds of coating agent were compared. Data are mean ± SE. *P < 0.05, vs. Matrigel. Mann-Whitney *U* test, n = 8–14. (**d**) Immunohistochemical staining for CK19 (bile duct endothelial cells, white), CD31 (endothelium, green), SE-1 (sinusoidal endothelial cell, green), CD26 (living tissue, Red) and HNF4α (hepatocyte, Red) of liver tissue on day 27. Scale Bar = 200 µm. (**e,f**) CD31- and HNF4α-positive cells in living cells were measured at day 27 after utilization of various coating agents before transplantation. The adult liver and fetal liver were determined as the control. Data are mean ± SE. *P < 0.05, vs. Matrigel Mann-Whitney *U* test, n = 3.

As mentioned, coating and fixing the transplanted tissue are necessary. In clinical application, the coating material must be a biological material that is safe for the living body. Considering that Matrigel does not fulfil the biological material standard (biogenic basis), we attempted to develop a biological coating agent that can replace Matrigel. The fetal liver tissue was transplanted on the liver surface 2 weeks after DMN administration. Matrigel, UPAL, fibrin, oxidised cellulose and sodium hyaluronate were used as coating agents on the liver surface. Two weeks after the transplantation, we sampled and histologically assessed the livers coated with different coating agents to investigate the engraftment area and graft survival of such livers (Fig. 3a). Engraftment of the transplanted tissue was confirmed by DPPIV staining, which also confirms the survival rate of transplanted tissue; in all of the coating groups, the engraftment rate was 75% or more (Fig. 3b). The engraftment rate was equal to the graft survival of transplanted fetal livers. However, in comparing the maximum area of the engrafted tissue by each coating agent, UPAL was the highest among the four coating types significantly, except Matrigel (Fig. 3c). Immunohistochemical staining was also performed to examine the ratio of the vasculature in the engrafted tissue and the degree of maturation of the liver tissue. Several markers in the engrafted tissue have been examined. These markers were the viable tissue marker (CD26), vascular endothelial cell marker (CD31), bile duct endothelial cell marker (CD19), hepatocyte marker (HNF4α) and sinusoidal endothelial cell marker (SE-1) (Fig. 3d). As a result, the ratios of HNF4α + area in the UPAL-coated group were almost equal to that in the Matrigel-coated group. In addition, the ratios of CD31 + area in the UPAL-coated group were significantly higher than those in other coating agents tested (Fig. 3e,f). In conclusion, UPAL was the most effective coating agent in terms of engraftment rate, engraftment area and the expression of liver function marker.

### Functional assessment of the transplanted fetal liver tissue in UPAL coating group

**Figure 4:**
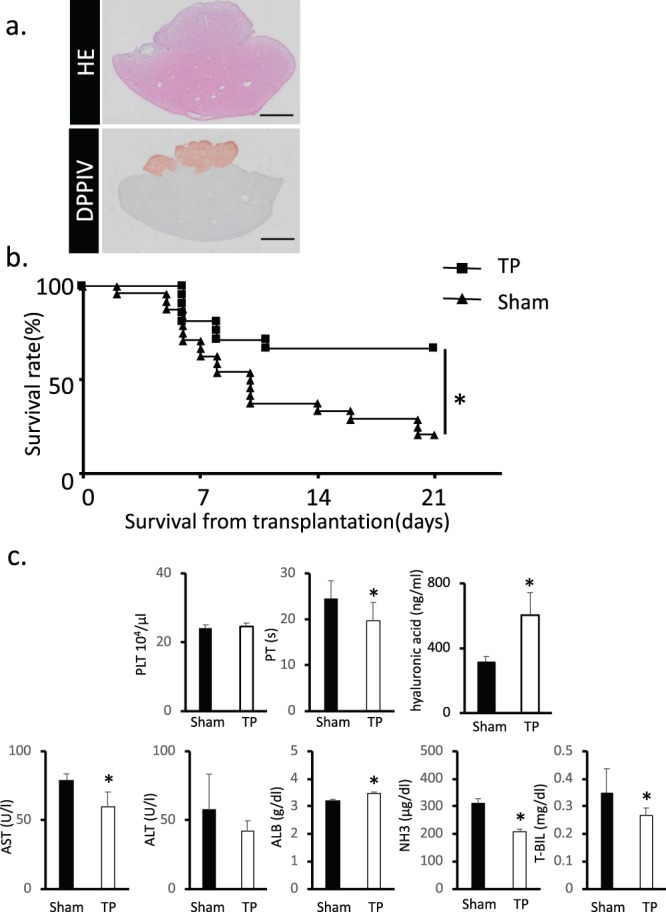
Functional assessment of the transplanted fetal liver tissue in UPAL coating group. (**a**) Histological analysis of the fetal liver tissue that was engrafted on the surface of the middle lobe of the liver and covered by UPAL. DPPIV-stained living tissue is shown in red. Scar bar: 5 mm. (**b**) Measurement of the survival rate of transplanted fetal liver tissue in UPAL group. The survival rate in the transplanted group without UPAL serves as the control. Data are shown as mean ± SEM. *P < 0.01, log rank test, Sham: n = 24, TP: n = 21. (**c**) Levels of PLT, PT, hyaluronic acid, AST, ALT, T-BIL, NH_3_ and ALB were also comparable between the sham and transplanted groups after UPAL coating. Data are mean ± SE. *P < 0.05, vs. sham Mann-Whitney *U* test, n = 6–9.

To assess the effect of the transplanted fetal liver tissue, we checked the engraftment rate of the graft tissue (Fig. 4a). The survival rate improved in rat fetal liver tissue transplanted to the liver surface compared with the sham operation group (Fig. 4b). The PT significantly decreased in fetal liver transplantation after UPAL coating, whereas the PLT level remained unchanged. The hyaluronic acid concentration in the transplanted group was higher than in the sham group. The expression levels of major liver function indices, namely, AST, ALB, T-BIL and NH_3_, in serum were significantly different between the sham and transplanted groups. In the engrafted group, these indices decreased, but the ALB expression increased. In the UPAL-coated liver surface group, blood biochemistry examination showed that the liver function indices AST and ALT level decreased after transplantation. Child-Pugh classification related markers, namely, T-Bil and NH_3_ levels, were improved. The serum hyaluronic acid concentration significantly increased after the coating transplantation compared with that in the sham group. In addition, the PT was shortened and the serum ALB level increased (Fig. 4c).

## Discussion

We understand that there are chemicals other than DMN that can be used to induce cirrhosis, including CCL4, TAA, and GalN^16^. However, compared to DMN, most of these chemicals either take a long time to induce cirrhosis, or are highly toxic to the animal^16^.

Regarding the death of the experimental animals, we dissected all the animals and found that the cirrhosis was the cause of death in the majority of the animals. Some of animals died of unknown causes, such as injury, infection, or anesthesia after the transplantation. Regarding the improvement of survival rate, we are considering modifying the dosage of DMN or using a DMN cocktail (together with other chemicals).

In recent years, many experimental liver cirrhosis therapies have been suggested and tested on the rodent models of fibrosis. However, all of these therapies have limitations, such as infarction of the liver by infused cells or poor engraftment of transplanted cells, in replacing the fibrotic tissues with normal liver tissues^17^. In addition, liver fibrosis might cause haemorrhage due to the decrease of PLT count and coagulation factor activity. Therefore, developing a minimally invasive and effective transplanting method is necessary for the treatment of liver cirrhosis. Recently, we have developed a novel liver cirrhosis therapy by transplanting fetal liver tissues onto the liver surface of cirrhosis.

As shown in our study, coating the surface of a cirrhotic liver with coating materials may gain higher engraftment area and survival rate of transplanted tissues than the noncoated cirrhotic liver (Fig. 2). We have tested several coating agents and found that both Matrigel and UPAL showed the best effect in enhancing the transplantation. Matrigel is a soluble and sterile extract of basement membrane proteins derived from the Engelbreth-Holm-Swarm tumor^18^. It contains several growth factors, such as basic fibroblast growth factor (bFGF), epidermal growth factor (EGF), insulin-like growth factor-1 (IGF-1), PLT-derived growth factor (PDGF), nerve growth factor and transforming growth factor-β (TGF-β)^19^. The HGF, EGF, TNF-α and IL-6 levels are related to liver regeneration^20–22^. EGF is one of the important factors in hepatocyte proliferation at an early stage in liver regeneration^23^. Meanwhile, the TGF-β, PDGF and IGF levels are involved in liver fibrosis. TGF-β plays an important role in suppressing hepatocyte proliferation and promoting liver fibrosis^24,25^. PDGF is a key factor in promoting the proliferation of hepatic stellate cells. It could be secreted by sinusoidal endothelial cells and activated stellate cells^26^. In our opinion, the growth factors contained in Matrigel may promote the growth and differentiation of the transplanted liver tissues, thereby increasing the engraftment area and survival rate. However, Matrigel is not suitable to be used for clinical application because this material might induce tumor growth^27^. Instead of Matrigel, we suggest that the UPAL gel is a more suitable coating material for clinical application in fetal liver tissue transplantation. Several reported examples showed that UPAL is safe to be used in biomedical and preclinical application^28–30^. Unlike Matrigel, the UPAL gel is a plant-derived polysaccharide matrix extracted from the cell walls of brown algae^28^. Thus, it might be free from potential animal pathogens. Furthermore, *in vivo* biological safety tests based on an international organization for standardization (ISO) and good laboratory practice standards showed that UPAL does not have immunogenicity to the human body^31^. In addition, the UPAL group provided more CD31- and HNF4α-positive cells than any other groups, illustrating that UPAL coating not only increased the engraftment area but also increased the expression of vascular endothelial cells and hepatocytes. Compared with other coating agents, UPAL yielded the best effect in improving transplant tissue survival rate and replenishing the liver function. Moreover, the intradiscal injection of UPAL gel can suppress lumbar intervertebral disc degeneration and promote the production of extracellular matrix, essential for nucleus pulposus cell function after discectomy^31^.

In addition, although it is general believed that alginate is non-biodegradable in mammals because of a lack of the corresponding enzyme, recent studies have showed opposite results. Indeed, recent reported have showed that UPAL gel disappears in rabbits at week 12, and in sheep at week 24, after transplantation, and no adverse effects were found in these animals^31^. Furthermore, it has been suggested that UPAL gel might progressively decline through physiological ion replacement (divalent to monovalent) and hydrolysis^31^.

In conclusion, we have improved the fetal liver tissue transplantation technology by coating the liver with UPAL. The results in this study proved that UPAL coating is feasible and safe and that it could greatly improve the success rate of liver organoid transplantation to the liver surface. The use of alginate as a wound healing dressing material has a number of advantages such as promoting functional expression of hepatocytes^32,33^. Moreover, UPAL gel could promote the repair of intervertebral disc and osteochondral defects^28–31^. Taken together, the UPAL coating of liver organoid might be a promising alternative method in liver transplantation for cirrhosis treatment.

This concept of UPAL coating might not only improve the success rate of fetal liver tissue transplantation but also produce a similar effect in the transplantation of other organs or tissues. In the future, studies about UPAL coating on the transplantation of other organs or tissues should be tested *in vivo*. Furthermore, follow-up studies are required to identify the mechanism of UPAL gel for the optimization of this material.

## Methods

### Isolation of fetal liver tissue in rats

ED14 fetal liver tissues were collected from the fetus of a pregnant DPPIV(+) F344 female rat (Japan SLC, Shizuoka, Japan) and used as donor tissues for transplantation. The rat fetus was sacrificed by euthanasia with isoflurane, and the fetal liver was removed under a microscope. The fetal liver tissue was immediately stored on ice in phosphate buffered saline (PBS) until transplantation. Liver tissue isolation time is critical; thus, the liver tissues must be isolated in minimal time.

### Induction of liver cirrhosis in rats

Three-week-old DPPIV(−) F344 rats (Japan Charles River, Kanagawa, Japan) were acclimated in an animal facility for 2 weeks before induction for liver cirrhosis. The animals were maintained under SPF environment with 12 h light and 12 h dark cycle. Moreover, we induced liver cirrhosis in these rats by intraperitoneal DMN injection at 10 mg/kg (body weight) concentration for 3 consecutive days/week for over 3 weeks. All animal experiments were designed according to the ethical rules of the Animal Research Center in Medical College of Yokohama City University. We bred and maintained the rats according to our institutional guidelines for the care and use of laboratory animals. The Institutional Animal Care Use Committee of Yokohama City University approved all our animal studies (Approval No. 17–25).

### Coating agents

Matrigel (Corning, New York, NY, USA), fibrin (Sigma-Aldrich, St. Louis, MO, USA), oxidised cellulose (Interseed; Johnson and Johnson, New Brunswick, NJ, USA)and sodium hyaluronate (Seprafilm; Kaken Pharmaceutical, Co, Ltd., Tokyo, Japan) were used as coating agents. Fibrin was freshly mixed with fibrinogen at 100:1 ratio before use. UPAL was kindly donated by Mochida Pharmaceutical co. ltd. Tokyo, Japan. Alginate was carefully placed over the transplanted tissue, then we add some calcium chloride to cover the alginate and prompt gelation.

### Tissue transplantation

First, the liver cirrhosis rat model was anaesthetized using isoflurane. Then, the abdominal wall of the animal was transected to expose the abdominal viscera. After blocking the portal blood flow using vascular clamp forceps, we sharpened the middle lobe surface by using an 18 G needle (Terumo corporation, Tokyo, Japan). After detachment, we performed compression hemostasis using a cotton swab. Then, transplanted the fetal liver tissue onto the DPPIV(−) F344 rats model’s cirrhotic liver surface. Meanwhile, the fetal liver tissues were isolated from the ED14 fetus of DPPIV(+) F344 pregnant rats. Next, 5 coating agents were separately applied over the transplanted fetal liver tissue. The size of coating agent application was similar to the transplantation site.

### Hematoxylin and eosin (HE) staining

For HE staining, we used the cryostat tissue sections (sections 1–4), which were first fixed with 10% formalin solution (FUJIFILM Wako Pure Chemical Corporation, Osaka, Japan), followed by incubation with Milli-Q water. Next, glass slides were deparaffinized thrice for 10 min in xylene and dehydrated with 100%, 95%, 80%, 70%, and 50% ethanol solutions. Then, it was replaced with Milli-Q water (paraffin tissue section). The slides were immersed in Carrazi’s hematoxylin (Muto Chemistry) for 10 min and rinsed with running tap water to remove excess stain from the slide. Afterward, the slides were immersed in eosin (Muto Chemistry) for 10 min and rinsed with Milli-Q water. Next, these slides were immersed in 50%, 70%, 80%, 95% and 100% ethanol solutions and then in xylene for 3 min. Such steps were repeated thrice. Thereafter, Mount-Quick (Daichi Sangyo Co., Ltd.) was dropped, and the sample was covered with a glass slide (Matsunami Glass).

### Immunohistochemical staining (frozen tissue section)

The frozen tissue sections were fixed with a solution containing a mixture of acetone and methanol in equal amounts. After air-drying, the dyed sample was surrounded by a water repellent pen (Dako). Then, permeabilization was performed thrice using 0.05% PBS with Tween 20 (PBST) for 10 min and then blocked at room temperature for 1 h using Blocking One (Nacalai Tesque). Thereafter, the primary antibody solution was diluted to an appropriate concentration with Blocking One and then stored at 4 °C overnight. After the reaction, the slide was washed thrice with PBST for 10 min. The secondary antibody solution was appropriately diluted with Blocking One (1: 500) and stored at room temperature for 1 h. After washing with PBS for 10 min, we added a mixture of 4′,6-diamidino-2-phenylindole dihydrochloride (DAPI, Invitrogen) and Apaci mounting agent (Wako) dropwise at 1:1000, covered with a glass slide (Matsunami Glass) and then sealed. Antibodies were as follows: CD26 (BD Bioscience 559639), CD31 (BD Bioscience 550300), CK19 (Progen Biotechnik GmbH, 61029), SE-1 (Immuno-Biological Laboratories, 10078) and HNF4 alpha (H-1) (Santa Cruz, sc-374229).

### DPPIV staining

Cryostat tissue sections were fixed with a solution containing a mixture of acetone and chloroform in equal volumes. Then, these sections underwent enzymatic histochemical staining with a staining solution for 20 min. The staining solution consisted of 1 mg/mL of Fast Blue BB Salt hemi (zinc chloride) salt (Sigma-Aldrich) in PBS and 8 mg/mL of Gly-Pro 4-methoxy-β-napthylamide hydrochloride (Sigma-Aldrich) in dimethyl sulfoxide (Wako), which were mixed at a ratio of 20:1 individually. Next, the stained sections were soaked in a 2% copper sulfate aqueous solution prepared by dissolving copper sulfate (II) pentahydrate (Wako) in Milli-Q water. To fix the tissue, we immersed the cells in 10% formalin for 10 min, followed by immersion with Milli-Q water and 10 min staining with Carrazi’s hematoxylin (Muto Chemistry). After rinsing with tap water, we washed the slides for 30 min under running tap water to remove excess stain. Finally, apical sealant (Wako) was dropped, and a glass slide (Matsunami Glass) was placed and sealed.

### Sirius red staining

Frozen and paraffin tissue sections were used for Sirius red staining. After fixation of frozen tissue sections using 10% formalin solution (Wako), it was replaced with Milli-Q water (frozen tissue section). The slides were deparaffinized thrice for 10 min in xylene and dehydrated with 100%, 95%, 80%, 70% and 50% ethanol solutions. Then, it was replaced with Milli-Q water (paraffin tissue section). The slides were immersed in 0.03% Sirius red/saturated picric acid solution (1% Sirius red solution saturated with aqueous solution picric acid) for 30 min and rinsed under Milli-Q water. Afterward, these slides were immersed in 50%, 70%, 80%, 95% and 100% ethanol solutions and then in xylene for 3 min. Such steps were repeated thrice. Finally, Mount-Quick (Daichi Sangyo Co., Ltd.) was dropped, and the sample was covered with a glass slide (Matsunami Glass).

### Measurement of liver function parameters

Liver functions were assessed by measuring several functional indicators in the blood. First, we collected blood from liver-transplanted rats at 14 post-transplantation days. The collected blood was anticoagulated with EDTA. Then, PLT and PT were determined in the blood samples by using Coagucheck® XS (Roche). To separate blood serum, we centrifuged the collected blood at 4,000 rpm for 20 min. The serum was used to evaluate liver function by measuring the liver function indices, namely, AST, ALT, T-BIL, NH_3_ and ALB. These indices were determined in the serum samples by using an automated clinical chemistry analyzer machine (DRI-CHEM 7000 V).

### Determination of hydroxyproline content in the liver

Total collagen was estimated by measuring the hydroxyproline, which is an amino acid characteristic of collagen. A portion of the sample was excised with a knife. The weight of the excised tissue was measured, the organ fragment was transferred to a biosmasher tube (Nippi), and four volumes of tissue weight 6 N HCl (Wako) was added and homogenized. The liver samples were hydrolyzed for 12–15 h in 5 mL of 6 N HCl at 96 °C. After removing from the heater and cooling at room temperature, these samples were centrifuged at 15,000 rpm for 5 min. Then, we collected an appropriate amount of the sample and added 0.5 volume of H_2_O. After preparing 20 μL of the sample, we added 75 μL of 50% 2-opropanol in citrate-acetate-buffered chloramine T. The mixture was vortex and mixed. Subsequently, we added 75 μL of a mixture of Ehrlich’s reagent (2-propanol (Wako), Dimethylaminobenzaldehyde (Sigma) and perchloric acid (Sigma). Tubes were incubated for 10 min in a water bath at 60 °C. The samples were placed on the range of 560 nm absorbance band in Hitachi spectrophotometer (U-2000). Results were expressed as μmol hydroxyproline/g of liver tissue.

### Statistical analysis

Data were presented as mean ± SE. The significance was analyzed by Mann-Whitney *U* test or log rank test using the GraphPad Prism 8 software. Moreover, *P* < 0.05 indicated statistical significance.

## Supplementary information

Video 1.
